# Influence of hip joint simulator design and mechanics on the wear and creep of metal-on-polyethylene bearings

**DOI:** 10.1177/0954411915620454

**Published:** 2016-05-09

**Authors:** Murat Ali, Mazen Al-Hajjar, Susan Partridge, Sophie Williams, John Fisher, Louise M Jennings

**Affiliations:** 1 Institute of Medical and Biological Engineering, School of Mechanical Engineering, University of Leeds, Leeds, UK; 2 Leeds Musculoskeletal Biomedical Research Unit, The Leeds Teaching Hospital NHS Trust, University of Leeds, Leeds, UK

**Keywords:** Hip replacement, hip simulator, polyethylene, wear

## Abstract

Hip joint simulators are used extensively for preclinical testing of hip replacements. The variation in simulator design and test conditions used worldwide can affect the tribological performance of polyethylene. The aim of this study was to assess the effects of simulator mechanics and design on the wear and creep of ultra-high-molecular-weight polyethylene. In the first part of this study, an electromechanical simulator and pneumatic simulator were used to compare the wear and creep of metal-on-polyethylene components under the same standard gait conditions. In the second part of the study, the same electromechanical hip joint simulator was used to investigate the influence of kinematics on wear. Higher wear rates and penetration depths were observed from the electromechanical simulator compared with the pneumatic simulator. When adduction/abduction was introduced to the gait cycle, there was no significant difference in wear with that obtained under the gait cycle condition without adduction/abduction. This study confirmed the influence of hip simulator design and loading conditions on the wear of polyethylene, and therefore direct comparisons of absolute wear rates between different hip joint simulators should be avoided. This study also confirmed that the resulting wear path was the governing factor in obtaining clinically relevant wear rates, and this can be achieved with either two axes or three axes of rotations. However, three axes of rotation (with the inclusion of adduction/abduction) more closely replicate clinical conditions and should therefore be the design approach for newly developed hip joint simulators used for preclinical testing.

## Introduction

Preclinical testing of hip joint replacements is essential for determining their safety and efficacy before being implanted into patients. The tribological performance of hip prostheses can be determined under different physiological kinetic and kinematic conditions using hip joint simulators.^1,2^ The development of tribological simulations has been based on the average patient performing a walking gait activity,^3–5^ initially undertaken to study the clinical failure of metal-on-polyethylene bearings^6,7^ where polyethylene (PE) wear and osteolysis leading to aseptic loosening and failure had been reported.^8^ This was the basis of the current international standard for preclinical hip wear simulation, ISO 14242-1:2014.^9^ Over the years, preclinical testing methods have been developed further to include a wider range of physiological conditions in the efforts to understand the reasons for increased clinical failure rates and achieve longer lasting implants that meet the demands of active patients. These enhanced preclinical testing methods include adverse conditions, where the variations in surgical implant positioning in 6 degrees of freedom are considered, and the changes in the implants such as damaged metal femoral heads and oxidative degradation of PE cups, as well as patient variation and activities have been considered.^10–13^ In order to assess the performance of hip joint replacements under adverse conditions, hip joint simulators that are capable of applying the necessary physiological kinetics and kinematics, and that comply with the latest international standards, are required.

The design and complexity of hip joint simulators and test conditions have varied greatly,^14–38^ meaning that comparing the results between different simulators has been challenging.^1,2^ Hip simulators should be capable of generating physiological wear paths and test conditions for relevant preclinical assessment.^28,29^ Pneumatic, hydraulic and electromechanical methods have all been used to apply mechanical loads and motions to the hip joint. Hip joint simulators require multiple stations for meaningful statistical comparisons to be carried out.

Ultra-high-molecular-weight polyethylene (UHMWPE) is used in the majority of hip prostheses, having been used for over 30 years with clinical success in the short- and medium term.^39–42^ Wear of PE has been one of the main factors limiting the successful long-term performance of metal-on-UHMWPE joint replacements.^7^ Ingham and Fisher^7^ were able to explain the resulting adverse biological response to PE wear debris, which led to osteolysis-induced aseptic loosening. To improve the clinical success rates of UHMWPE, there has been a continuous effort to improve wear resistance. Hip joint simulators using standard conditions were successful at predicting the improvement in wear resistance of cross-linked PE compared to conventional PE, which was reflected in clinical studies.^43–45^ The large variation in the wear of metal-on-UHMWPE reported clinically^43,46,47^ has not been replicated in vitro, in studies that have applied the standard walking cycle assuming an average patient with a well-positioned implant.

The cross-shear effects on the wear of PE are well understood and have been demonstrated experimentally and computationally.^48–50^ Therefore, there is a need to simulate multi-directional elliptical sliding contact paths during the gait cycle.^25^ The wear rates of conventional PE from hip simulators were reported in the literature to be 35–50 mm^3^/million cycles.^39,51^ Improved wear resistance and reduction in the cross-shear effects were found with the introduction of cross-linked PE. Wear rates below 20 mm^3^/million cycles were reported for moderately cross-linked PE and even less for highly cross-linked PE.^51,52^ Cross-linked PE has now shown over 10 years of clinical success with reduced clinical wear rates.^53,54^

Wear of PE occurs as a result of predominantly sliding contact with the counterface during articulating motion of the hip joint. Creep is the permanent deformation of PE due to loading conditions. Both wear and creep of PE were measured using hip joint simulators,^20,51,52,55^ where higher penetration depths were observed before steady-state wear rates were reached. This explains the higher short-term linear penetration depths measured in vivo following implantation.^43^ Both wear and creep are observed clinically; therefore, assessing both simultaneously in the laboratory has become an integral part of preclinical hip simulation.

The first-generation ProSim pneumatic hip joint simulator has been used extensively over the past 15 years to determine wear of hip prostheses under standard gait conditions. This simulator applies a single axis of loading pneumatically and has two independently controlled axes of motion, flexion/extension (F/E) and internal/external (I/E) rotation, which are applied electromechanically. The standard gait cycle is run with the phase angle of the I/E rotation 90° out of phase with the F/E motion. This configuration has been shown to give a physiologically relevant biaxial wear path between the articulating surfaces, producing wear results similar to those observed in vivo.^10^

In order to develop enhanced preclinical wear simulation methods that investigate a wider range of clinical and patient conditions, be it surgical positioning or higher patient activity levels, as well as meet the requirements of international standards, it is necessary to develop and design hip simulators to meet these requirements. In this study, a new electromechanical hip simulator is presented that is able to meet the requirements of the international standard and perform testing under adverse conditions. In the first part of this study, wear rates of metal-on-moderately cross-linked UHMWPE bearings tested using this newly developed electromechanical hip joint simulator ProSim EM13 (‘EM’ stands for ‘electromechanical’ and ‘13’ stands for the year of commissioning) were compared with those obtained from the pre-existing ProSim pneumatic hip joint simulator. Second, the influence of kinematics on the wear of moderately cross-linked UHMWPE was investigated through comparison of two axes and three axes of rotation conditions with different phasing using the same electromechanical hip simulator.

## Materials and methods

The wear of ten 36-mm-diameter metal-on-polyethylene (Marathon™; DePuy Synthes Joint Reconstruction, Leeds, UK) hip replacements was determined using ProSim EM13 (n = 6) and ProSim pneumatic (n = 4) hip simulators (Simulation Solutions, Stockport, UK). In both simulators, the acetabular cup (liner) was placed in an anatomical position, and to avoid the complexity of angled stems, the femoral head components were secured onto tapered vertical spigots fixed onto a holder. On the electromechanical simulator, the cup inclination angle was set to 30° to the horizontal plane which complies with the latest international standard and is equivalent to 40° in vivo. To be consistent with previous testing carried out on the pneumatic simulator, the cup inclination angle was set to 35° to the horizontal plane, which is equivalent to 45° in vivo.^56,57^ The 5° difference in cup inclination angle between the two simulators would contribute to the different wear zone locations; however, no effect on wear rates or penetrations depths was anticipated. These cup inclination angles were at or below 45° where the wear rates of PE are expected to be the same,^58^ since the wear area was well within the bearing surfaces and there was only 5° difference between the two groups. The acetabular cups were mounted into appropriate metallic shells cemented within a cup holder, which allowed removal for gravimetric measurement purposes.

**Figure 1.**
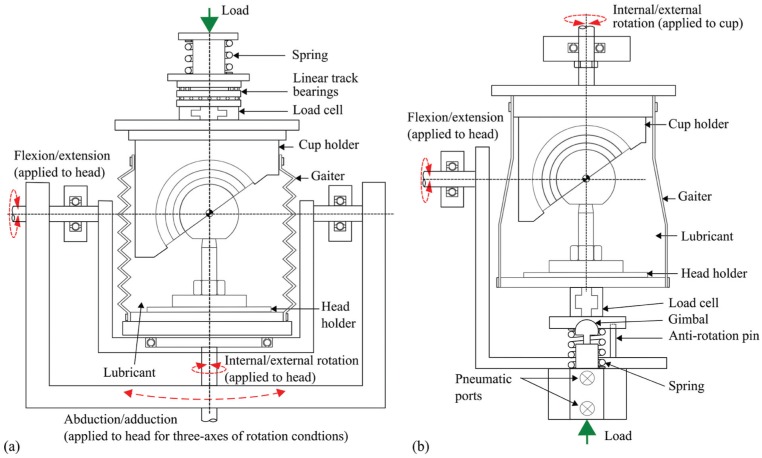
Test cell schematics of (a) the electromechanical hip joint simulator and (b) the pneumatic hip joint simulator.

On the electromechanical simulator (Figure 1(a)), the load was applied in a vertical direction (along the superior/inferior axis) through the centre of the acetabular cup through a spring by a camshaft during the gait cycle. The cup holder was attached to an assembly with two sets of linear track bearings to allow for passive medial/lateral and anterior/posterior displacement; therefore, any misalignment was corrected by allowing the cup to self-centre. All angular displacements were applied to the femoral head about its centre of rotation.

On the pneumatic simulator (Figure 1(b)), the load was applied pneumatically through the centre of the femoral head in the direction normal to the F/E rotation. The angular displacements on the pneumatic simulator were applied using motors. The I/E rotation was applied to the acetabular cup and F/E rotation was applied to the femoral head. Each station on the ProSim pneumatic simulator had a gimbal below the femoral head holder, which allowed the femoral head to be self-centred.

**Figure 2.**
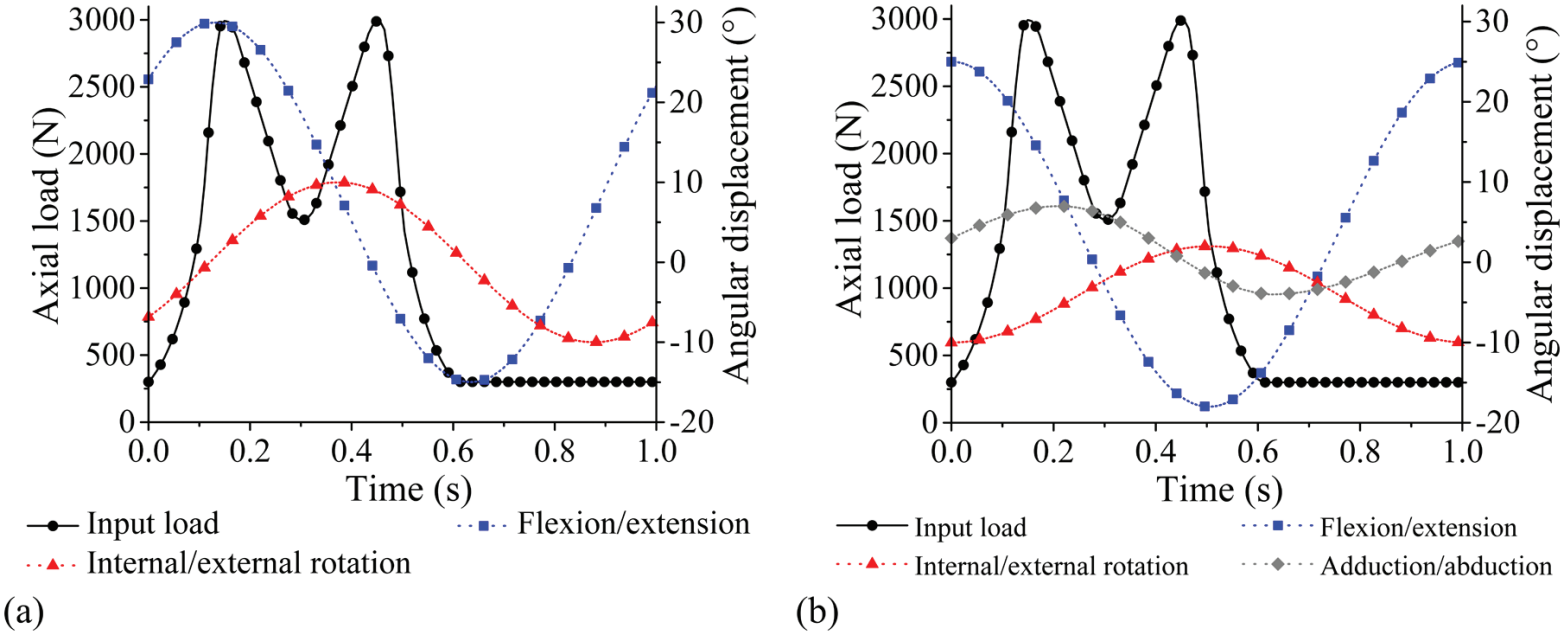
Simulator input profiles: (a) load and angular displacements for two axes of rotation conditions and (b) load and angular displacements for three axes of rotation conditions.

A twin-peak input load profile was applied for all tests to obtain peak output loads of 3 kN and a swing phase load 0.3 kN (Figure 2(a)). F/E (+30°/−15°) and I/E rotation (±10°) was applied for the two axes of rotation conditions. The phase angle of the I/E rotation was 90° out of phase with the F/E motion, consistent with previous studies,^10,29^ in order to generate a biaxial wear path. The test ran for a total of 5 million cycles for each simulator with measurement intervals at 1, 2, 3 and 5 million cycles.

For the second part of the study, three axes of rotation were applied following the international standard ISO 14242-1:2014 (Figure 2(b)),^9^ therefore including adduction/abduction of +7°/−4° within the standard gait cycle. The three axes of rotation simulation were completed on the electromechanical simulator and ran for 3 million cycles with measurement points at 1, 2 and 3 million cycles.

Load soak control samples (n = 2 for ProSim EM13, n = 1 for ProSim pneumatic) were used to measure the creep deformation of the PE liners and un-loaded soak control samples (n = 3 for ProSim EM13, n = 1 for ProSim pneumatic) were used to measure the weight change due to fluid absorption. The lubricant was 25% new-born calf serum diluted with deionised water (v/v) which was supplemented with 0.03% sodium azide (v/v) to retard bacterial growth. The serum was replaced approximately every 330,000 cycles.

At each measurement interval, the cups were removed from the simulator component holders, cleaned and allowed to stabilise in a humidity- and temperature-controlled environment for at least 48 h prior to measurement. The change in mass was determined using a microbalance (Mettler Toledo XP205 analytical balance, Greifensee, Switzerland) and subsequently converted into volumetric wear using a density of 0.934 × 10^−3^ g/mm^3^ for UHMWPE. A coordinate measurement machine (CMM; Legex 322, Mitutoyo, Japan) was used to measure the penetration due to wear and creep through construction of a three-dimensional map of the acetabular liner surfaces and analysis using Redlux software (Southampton, UK). The worn components were compared to pre-test measurements to determine the location of the wear zone and maximum penetration depths of the test samples and the loaded soak control samples. The mean wear rates were calculated with 95% confidence limits. One-way analysis of variance (ANOVA) and a paired sample t-test were carried out for statistical analysis as appropriate. Significance levels were taken at p < 0.05 indicating a difference statistically between groups.

## Results

**Figure 3.**
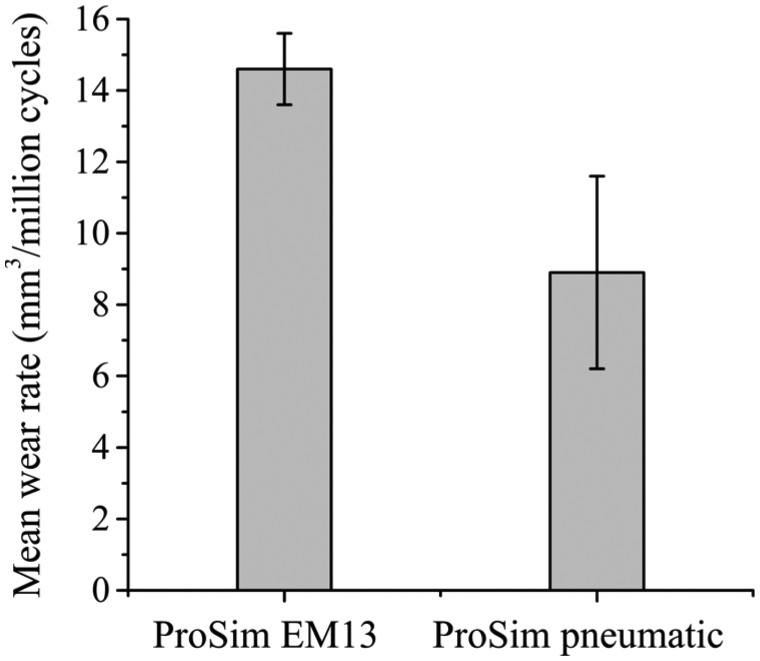
Mean wear rates of UHMWPE from 0 to 5 million cycles using electromechanical and pneumatic simulators. Error bars represent ±95% confidence limits.

**Figure 4.**
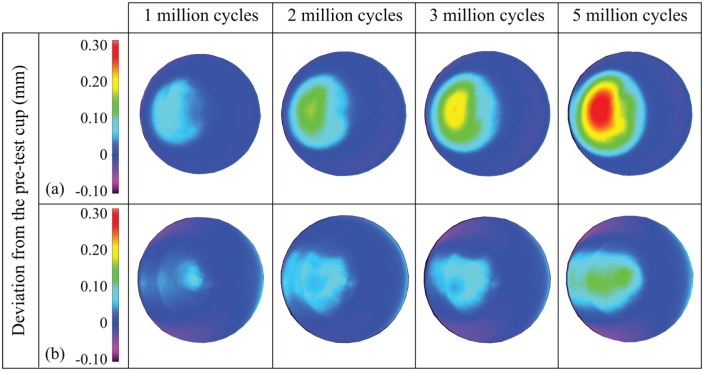
Three-dimensional representation of the wear and creep of polyethylene liners from 1 to 5 million cycles, obtained using the CMM and Redlux software, after testing on (a) the electromechanical simulator and (b) the pneumatic simulator. Positive values indicate penetration on the liners.

**Figure 5.**
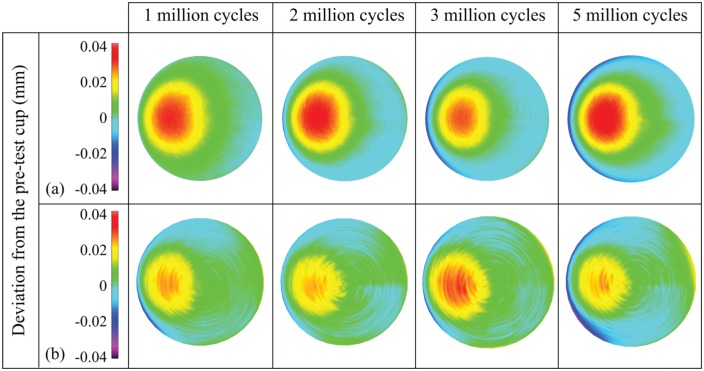
Three-dimensional representation of the creep of the loaded soak control polyethylene liners from 1 to 5 million cycles obtained using the CMM and Redlux software, after testing on (a) the electromechanical simulator and (b) the pneumatic simulator. Positive values indicate penetration on the liners.

**Figure 6.**
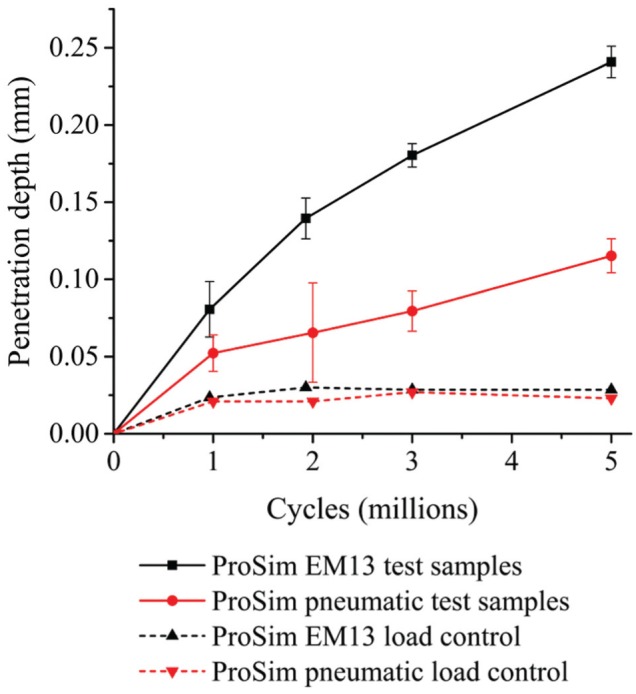
Maximum penetration depth of polyethylene liners from 0 to 5 million cycles (mean ±95% confidence limits).

The overall (0–5 million cycles) mean wear rates (±95% confidence limits) of the metal-on-UHMWPE bearings tested in the electromechanical and pneumatic simulators were 14.6 ± 1.0 and 8.9 ± 2.7 mm^3^/million cycles, respectively (Figure 3). Between 0 and 5 million cycles, the mean wear rate from EM13 was significantly higher (p < 0.01) compared with the pneumatic simulator. The combined wear and creep penetration depth increased at each measurement point for both electromechanical and pneumatic simulators (Figure 4). The penetration depth due to creep was determined from the loaded soak control components, where similar creep was observed using both simulators (Figure 5). The maximum mean wear and creep penetration depth from 0 to 5 million cycles for each test sample and loaded soak control sample was measured (Figure 6). The penetration depth increased at each measurement interval, and at 5 million cycles the mean maximum penetration depths (±95% confidence limits) measured for the electromechanical and pneumatic simulators were significantly different at 0.24 ± 0.01 and 0.12 ± 0.01 mm, respectively (p < 0.01). The loaded soak control samples with no articulation showed that the majority of the PE creep occurred within the first million cycles of testing.

**Figure 7.**
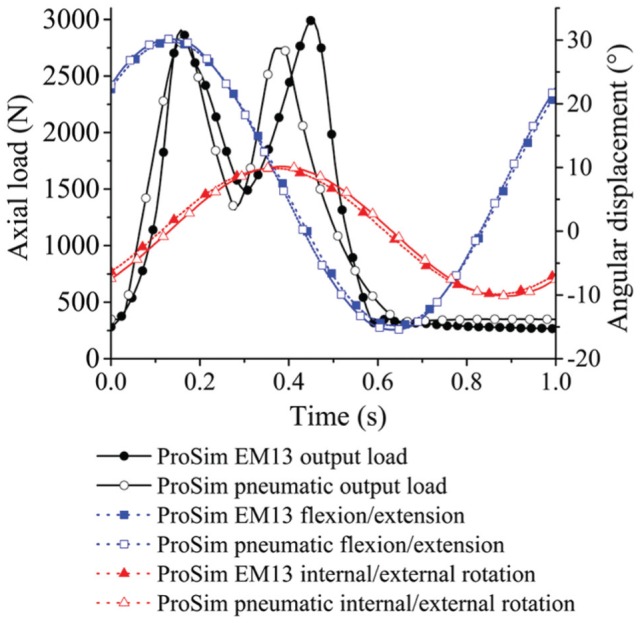
Typical output load and motions from the electromechanical and pneumatic simulators.

Differences in typical output load profiles from the electromechanical and pneumatic simulators across the gait cycle were observed. The main differences between the electromechanical and pneumatic simulators were the phasing and magnitude of the peak loads during the gait cycles and the transition rate from the second peak load to the swing phase load (Figure 7). A similar pneumatic output load profile was reported in a previous study by Liu et al.^59^ The electromechanical simulator was able to more closely match the prescribed input load because the motor was able to respond rapidly to the variation in load compared with the pneumatic system. The output angular displacement profiles from both simulators closely matched the input profiles (Figure 2a, Figure 7). Similar output F/E and I/E rotation motions were observed from both simulators (Figure 7).

**Figure 8.**
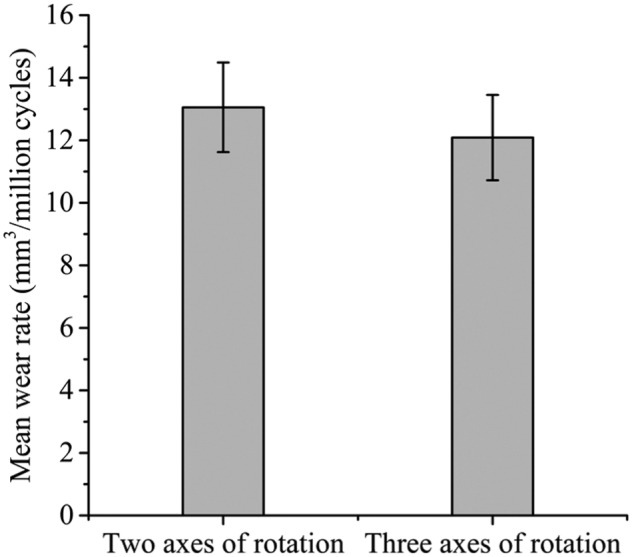
Wear rates of 36-mm metal-on-UHMWPE after 3 million cycles of testing using the electromechanical simulator under two axes and three axes of rotation conditions (mean ±95% confidence limits).

The mean wear rates over 3 million cycles of testing under the two axes and three axes of rotation conditions in the electromechanical simulator were 13.1 ± 1.4 and 12.2 ± 1.4 mm^3^/million cycles, respectively (Figure 8). There was no significant difference (p = 0.32) between the mean wear rates of the metal-on-UHMWPE bearings using two axes and three axes of rotation.

## Discussion

This study has used two different simulator designs to study the effects of simulator mechanics and kinematics on the wear and creep of cross-linked PE. Different wear rates, geometric wear and penetration depths of moderately cross-linked PE liners were observed using different hip joint simulators when applying the same kinematic input profiles. Applying I/E rotation to the acetabular cup and F/E to the femoral head on the pneumatic simulator compared to both motions applied on the femoral head on the electromechanical simulator may have contributed to the different wear rates from the simulators.^60^ Unconstrained passive medial/lateral and anterior/posterior displacement of the acetabular cup on the electromechanical simulator is thought to be another contributing factor. On the pneumatic simulator, any misalignment of the head and cup bearing centres was corrected with a gimbal arrangement allowing two axes of rotation of the head rather than linear displacement of the cup. The mechanics of a moving load vector applied to the femoral head on the pneumatic simulator is different to a fixed load vector applied through the acetabular cup on the electromechanical simulator. The variations in loading profiles may also have contributed to different wear rates between simulators.

Similar creep deformation and creep penetration were observed between the loaded-only samples from both simulators. These loaded soak control samples have confirmed that under cyclic loading conditions, the PE liners reach maximum creep deformation between 1 and 2 million cycles, as observed in previous experimental, computational and clinical studies.^43,52,59^ The magnitude of penetration due to creep was found to be similar to that estimated clinically.^61^

Designing a hip joint simulator with three axes of rotation conditions can replicate clinical hip joint motion and allow compliance with the latest ISO standards. However, the simplicity of a simulator with two axes of rotation is still capable of applying clinically relevant wear paths with the correct phasing and magnitude of each rotational axis as discussed by Barbour et al.^29^ In this study, similar wear rates of PE under two axes of rotation conditions compared with full three axes of rotation conditions were obtained, further validating Barbour et al.’s study. However, it should be noted that this was based on standard testing conditions only. The test conditions used in this study are expected to represent conforming hip bearing contact leading to idealised two body wear.^6^ The wide envelope of conditions in vivo including the variations in surgical positioning of the hip joint implant, prosthetic design, patient activities and conditions^62–64^ may lead to a wider variation in wear scars to those produced by the standard conditions used in the simulators in this study.

This study has confirmed the importance of designing simulators with load control stations to determine the creep deformation of PE for geometric assessments. This does not fully replicate dynamic creep of articulating surfaces; however, it is a practical way of monitoring the creep deformation while avoiding sliding contact between the femoral head and acetabular cup. Wear rates of conventional PE have been significantly reduced with the introduction of moderately and highly cross-linked PE. Therefore, it is necessary to use soak samples to monitor the level of fluid absorption during a test^55,65,66^ and to correct gravimetric measurements masked by fluid absorption.

Although comparison of wear rates between simulators can offer an initial form of validation for newly designed simulators, direct comparison of wear rates should be avoided. It is crucial to understand the design and mechanics of the simulators used for in vitro testing and ensure clinically relevant hip joint motion is replicated. Two-axis simulators have been used in previous studies because of their simplicity compared with three-axis simulators; however, the need for advanced preclinical testing methods^62–64^ has led to the design of more complex simulators. The application and control of hip joint load and motion using motors provided consistent and highly conforming output to input profiles; therefore, it is a recommended solution for the future design of hip simulators. Higher accuracy and precision of output loads from the electromechanical simulator compared with the pneumatic simulator and the inclusion of adduction/abduction rotation for full three axes of rotation are important considerations for simulators used for future preclinical testing.

Future studies will consider the effects of kinematics under two axes and three axes of rotation conditions with adverse microseparation conditions.^67,68^ Testing hip replacements under these conditions is one example stated in the stratified approach for enhanced reliability (SAFER) to meet the current and future demands of safer and more reliable hip joint replacements.^62–64^ These approaches justify the importance of designing and understanding current and future in vitro testing methods for preclinical testing.
